# Assessment of Frontal Hemispherical Lateralization in Plaque Psoriasis and Atopic Dermatitis

**DOI:** 10.3390/jcm12134194

**Published:** 2023-06-21

**Authors:** Szabolcs Bozsányi, Natália Czurkó, Melinda Becske, Roland Kasek, Botond Keve Lázár, Mehdi Boostani, Fanni Adél Meznerics, Klára Farkas, Noémi Nóra Varga, Lili Gulyás, András Bánvölgyi, Bence Ágoston Fehér, Emese Fejes, Kende Lőrincz, Anikó Kovács, Hunor Gergely, Szabolcs Takács, Péter Holló, Norbert Kiss, Norbert Wikonkál, Imre Lázár

**Affiliations:** 1 Department of Dermatology, Roswell Park Comprehensive Cancer Center, Buffalo, NY 14263, USA; 2 Department of Dermatology, Venereology and Dermatooncology, Semmelweis University, 1085 Budapest, Hungary; czurkonatalia@gmail.com (N.C.); lazar.keve.botond@gmail.com (B.K.L.); mehdi_parsii@yahoo.com (M.B.); meznerics.fanni@stud.semmelweis.hu (F.A.M.); farkas.klara@phd.semmelweis.hu (K.F.); varga.noemi@stud.semmelweis.hu (N.N.V.); gulyaslili1998@gmail.com (L.G.); banvolgyi.andras@med.semmelweis-univ.hu (A.B.); lorincz.kende@med.semmelweis-univ.hu (K.L.); kovacs.aniko@med.semmelweis-univ.hu (A.K.); gergely.hunor@med.semmelweis-univ.hu (H.G.); hollo.peter@med.semmelweis-univ.hu (P.H.); kiss.norbert@med.semmelweis-univ.hu (N.K.); wikonkal.norbert@med.semmelweis-univ.hu (N.W.); 3 Institute of Behavioral Sciences, Semmelweis University, 1089 Budapest, Hungary; becske.melinda@gmail.com (M.B.); roland@kasek.com (R.K.); agostonclub@gmail.com (B.Á.F.); 4 Selye János Doctoral College for Advanced Studies, 1085 Budapest, Hungary; mesifejes@gmail.com; 5 Institute of Psychology, Faculty of Humanities, Károli Gáspár University of the Reformed Church in Hungary, 1042 Budapest, Hungary; takacs.szabolcs@kre.hu; 6 Central Hospital of Northern Pest–Military Hospital, 1139 Budapest, Hungary; 7 Institute of Social and Communication Sciences, Faculty of Humanities and Social Sciences, Károli Gáspár University of the Reformed Church in Hungary, 1091 Budapest, Hungary

**Keywords:** psychodermatology, atopic dermatitis, plaque psoriasis, hemispherical lateralization, electroencephalography, heart rate variability, psychometry, metacognition

## Abstract

Background: Each brain hemisphere plays a specialized role in cognitive and behavioral processes, known as hemispheric lateralization. In chronic skin diseases, such as plaque psoriasis (Pso) and atopic dermatitis (AD), the degree of lateralization between the frontal hemispheres may provide insight into specific connections between skin diseases and the psyche. This study aims to analyze the hemispherical lateralization, neurovegetative responses, and psychometric characteristics of patients with Pso and AD. Methods: The study included 46 patients with Pso, 56 patients with AD, and 29 healthy control (Ctrl) subjects. The participants underwent frontal electroencephalogram (EEG) measurement, heart rate variability (HRV) assessment, and psychological tests. Statistical analyses were performed using ANOVA, with Bonferroni correction applied for multiple comparisons. Results: This study shows a significant right-lateralized prefrontal activity in both AD patients (*p* < 0.001) and Pso patients (*p* = 0.045) compared with Ctrl, with no significant difference between the AD and Pso groups (*p* = 0.633). AD patients with right-hemispheric dominant prefrontal activation exhibited increased inhibition and avoidance markers, while Pso patients showed elevated sympathetic nervous system activity. Conclusion: Psychophysiological and psychometric data suggest a shared prevalence of right-hemispheric dominance in both AD and Pso patient groups. However, the findings indicate distinct psychodermatological mechanisms in AD and Pso.

## 1. Introduction

The investigation of the psychological underpinnings of diseases has been a long-standing area of interest for medical practitioners and researchers. However, the study of this subject matter requires significant expertise and is particularly challenging. Among this field’s many intriguing and intricate facets is examining the psychological factors contributing to skin diseases. Over the course of several decades, it has been observed that left-handed patients are more prone to allergic diseases [1]. Additionally, research has shown that right hemispherical dominance can lead to adverse immune responses in both animals and humans. This is because the brain’s two hemispheres have been found to act differentially on behavior and immunity [2]. While acute stress can be advantageous to the body, aiding in adaptation and survival in “fight or flight” situations, chronic stress leads to the persistent activation of stress–response axes. This is associated with numerous autoimmune and chronic inflammatory diseases [3,4].

Atopic dermatitis (AD) is the most prevalent chronic inflammatory skin disease [5,6]; the point prevalence of the disease ranges from 2.7% to 20.1% in children [7] and from 2.1% to 4.9% in adults [8]. AD is characterized by impaired epidermal barrier function, sensitive and dry skin, eczematous lesions that can be localized or disseminated, and intense itching. These characteristics present differently by age, severity, and ethnic background [9,10], with about 80% of cases manifesting in infancy or childhood and the remaining cases developing in adulthood. 

While early studies suggested that a Th2 deviation was the primary factor contributing to the disease, subsequent research has highlighted a subgroup of patients with atopic dermatitis (AD), who exhibit a genetically predetermined, impaired epidermal barrier as the leading cause [11]. The pathogenesis of AD is attributed to the failure of keratinocyte differentiation and strong type 2 immune responses, with additional activation of the Th22, Th17/IL-23, and Th1 cytokine pathways [12]. The disease can be characterized by both Th1 activity and a transitory predominance of Th2 activity. Their dominance varies, and this appears in the context of the sequential cell activation of Th2 and Th1, the dynamics of which depend on the chronicity of atopic dermatitis [13]. Despite this, many immune pathways associated with the disease remain to be fully elucidated. Moreover, AD is associated with increased rates of depression and anxiety, which may create a vicious cycle of stress caused by the disease and worsening symptoms triggered by stress [14,15]. This is also true in reverse, and psychosocial stresses, such as anxiety or acute life events, can worsen AD and cause acute exacerbations [16,17,18]. Furthermore, according to recent studies, stress can induce the release of various neuroendocrine mediators, such as adrenocorticotropin, β-endorphin, catecholamines, and cortisol, which can weaken the function of the skin barrier, making it more susceptible to inflammatory conditions, such as AD [17,19]. Furthermore, a recent study showed that AD was associated with lower resilience to stress, indicating a higher risk of adverse psychiatric and somatic outcomes later in adulthood [20]. Nevertheless, the psychophysiological background of AD requires further elucidation.

Plaque psoriasis (Pso) is a chronic, immune-mediated inflammatory disorder that primarily affects the skin and joints, and is characterized by a primary inflammatory cell disorder with subsequent keratinocyte proliferation [21]. The disease affects approximately 2–3% of the world’s population, with varying prevalence rates in different regions [22]. Lesions typically appear as well-defined, erythematous, itchy papules and plaques covered with dry, white, or silvery scales, commonly on the skin of the elbows, knees, lumbosacral region, and scalp. However, they can appear anywhere on the body. Pso patients typically face psychosocial challenges, including anxiety, depression, and social avoidance due to concerns over others’ reactions to their disease, resulting in avoidance behavior and excessive worry. In addition, recent studies suggest that these patients exhibit cognitive biases toward self-referential or socially evaluative information [23]. Although the relationship between stress and Pso exacerbation has been explored in retrospective studies, the evidence for a strong association remains inconclusive [24].

The pathogenesis of Pso is multifactorial and has yet to be fully elucidated. Evidence suggests that genetic predisposition and environmental factors, such as infections, trauma, drugs, alcohol, smoking, and stress, interact to trigger the disease [25,26]. Psoriatic inflammation is mediated by innate and adaptive immune responses involving various cells and cytokines [27,28].

Psychological factors, such as stress, depression, and anxiety, can contribute to the development and exacerbation of Pso [29,30,31]. Similarly, as in AD, these factors can affect the immune system and inflammation levels, which may affect its onset and progression. In total, 31–81% of patients report stress as a trigger for their Pso [29]. Additionally, Pso and psychological background are in a circular, causal relationship. Individuals with Pso may also experience symptoms of depression and anxiety due to their disease. One in thirty-nine patients with severe Pso or one in eighty-seven patients with mild Pso may develop depression over one year [32]. A multicenter cross-sectional study reported increased incidence of suicidal ideation in patients with Pso compared with the general population and a lack of adequate diagnosis of depression in such patients [33]. Addressing these psychological factors may help to improve the overall well-being of individuals with Pso. Stressful life episodes often precede exacerbations of Pso episodes [34], and lesions occurring on hands or feet are difficult to treat [35]. Despite the complexity of the relationship between the skin and the mind, several studies have shown promising results in analyzing the interrelation between the two [36].

The relationship between hemispheric lateralization, emotional-motivational tendencies, and emotional reactivity has been studied for several decades [37,38]. Stronger relative left prefrontal activity is associated with positive emotions and approach motivation, while increased right prefrontal activity is associated with negative emotions and avoidance motivation [39]. 

Frontal electroencephalogram (EEG) asymmetry refers to the quantification of the distinction in EEG alpha power observed between corresponding right and left frontal electrodes [40]. The relative left frontal asymmetry (rLFA) value of a frontal EEG at 8–13 Hz reflects the left and right frontal hemispherical activity ratio, where lower values indicate a shift toward right-hemispheric-dominant prefrontal-predominant rLFA values below zero; we are talking about a dominant right-hemispheric prefrontal activation or relative right prefrontal dominance. As alpha activity has an inhibitory influence on cortical network activity, lower frontal asymmetry scores (right minus left alpha) reflect less left than right cortical activity (rLFA relative to left frontal activity) = log (right alpha activity)–log (left alpha activity) [41].

Increased rightward frontal alpha asymmetry suggests relatively increased right frontal activity, previously linked with anxiety disorders and depression. Crost, Pauls, and Wacker examined the relationship between anxiety and rLFA [42]. Those with higher anxiety had lower rLFA under situations of social threat.

Components of the Behavioral Inhibition System (BIS) and Behavioral Activation System (BAS) have been related to prefrontal cortex structures. A relative increase in left hemisphere activity was observed with positive emotional stimuli. In contrast, greater right hemisphere activity was associated with negative emotions [43], and more intense right hemispherical bias was associated with inhibition and avoidance.

BIS reflects the sensitivity to punishment that promotes the negative reinforcement of avoidance and withdrawal behavior. The left prefrontal area is related to motivations and emotions, while the right-hemispheric-dominant prefrontal activation relates to behavioral inhibition characteristics. BIS and BAS psychometry explores the personal behavioral features of the behavioral inhibition versus activation system. Metacognition and cognitive capacity have been tested in short-term memory experiments of increasing complexity. 

The dominant tendencies of sympathetic–parasympathetic activation can be excellently assessed by heart rate variability (HRV), a measure of stress management. With the significant role of metacognition in reasoning, empathy, theory of mind, and adaptive coping strategies, it is crucial to investigate its interplay in the pathogenesis of dermatological illnesses with psychological backgrounds [44]. Furthermore, as neurolinguistic therapy and psychotherapy require metacognitive abilities [45], understanding how and to what extent control (Ctrl) and experimental groups differ in this regard is essential for developing effective therapeutic interventions, quality-of-life improvement processes, and wellness programs.

Our study comprehensively investigated the hemispherical lateralization and the neurovegetative parameters, including EEG and HRV. We aimed to gain a deeper understanding of the complex psychological background of skin diseases by exploring these physiological and neurobiological factors in relation to these conditions.

## 2. Materials and Methods

### 2.1. Study Participants

This study was carried out at the Department of Dermatology, Venereology, and Dermatooncology of Semmelweis University. A total of 131 participants were recruited, comprising 46 patients diagnosed with Pso, 56 patients with AD, and 29 healthy volunteers without skin disease. The severity of the disease was assessed using a 5-point Investigator’s Global Assessment (IGA) score, where 1 indicated almost clear and 5 indicated very severe [46]. As the age of the participants could also influence the analyzed variables, the participants were split into two groups based on their age: under 40 years of age and above 40 years of age. Before participation, all subjects provided informed consent.

### 2.2. EEG Recording

The Hypnodyne Zmax^®^ headband manufactured by Hypnodyne Corp was utilized to record brain electrical activity. EEG signals were recorded from bilateral and lateral frontal channels, specifically F7 and F8. The EEG signals were sampled at a frequency of 256 Hz, and our analysis focused on alpha waves within the frequency range of 8–13 Hz. The lateralization index was calculated and used to indicate the relative dominance of the left hemisphere. Higher values of the lateralization index indicated lower right hemisphere dominance.

The alpha power was calculated using the fast Fourier transform method with zero padding. We employed two-second windows with 50% overlap within instrument-free sections of the recorded EEG signals to calculate the alpha power. The total duration of these sections was approximately eight minutes. 

### 2.3. HRV Measurement

We measured the HRV, sympathetic nervous system (SNS), and parasympathetic nervous system (PNS) activity using Cardiax^®^ version 4.41.1 (IMED Co Ltd., Budapest, Hungary) and subsequently evaluated the data using Kubios^®^ 2.1 software (BSAMIG, Biosignal Analysis, and Medical Imaging Group). We used the PNS index, SNS index, and root-mean-square of successive differences (RMSSD) to assess the HRV.

### 2.4. Psychological Questionnaires

Several psychological questionnaires assessed various aspects of the subject’s mental health. The Relationship Scale Questionnaire for Adult Attachment (RSQAA) [47], Parental Bonding Instrument (H-PBI) [48], and Adverse Childhood Experiences (ACE) [49] Questionnaire were used to measure attachment style, parental treatment, and childhood trauma, respectively. The BIS and BAS Scale [50] were used to evaluate the functioning of systems that activate and inhibit behavior. At the same time, the Hungarian version of the Brief COPE Questionnaire (BACQ) scales [51] was employed to measure coping style. In addition, a metacognition test was performed [52] to assess self-awareness, self-reflection, and other autognostic features of the human mind.

### 2.5. Statistics

Data analysis was performed using a two-way analysis of variance (ANOVA) test, with Bonferroni’s post hoc test to examine the differences between groups. Differences were considered significant at *p* < 0.05, and the homogeneity of variance between groups was assessed using the Levene test. The statistical software SPSS v.27 (SPSS Inc., Chicago, Ill., USA) was utilized for these calculations.

Owing to measurement difficulties in the rLFA and the HRV, different numbers of people were represented. To account for the potential effect of age on the variables analyzed, the participants were divided into two groups: those under 40 and those above 40. Specifically, the rLFA research included 11 Ctrl subjects, 26 individuals with AD, and 4 individuals with Pso (total of 41 participants) who were under 40 years of age, while 7 Ctrl subjects, 14 individuals with AD, and 18 individuals with Pso (total of 39 participants) were above 40 years of age. Similarly, in the HRV research, 13 Ctrl subjects, 36 individuals with AD, and 8 individuals with Pso (totaling 57 participants) were under 40 years of age, while 4 Ctrl subjects, 18 individuals with AD, and 33 individuals with Pso (totaling 55 participants) were above 40 years of age. 

## 3. Results

### 3.1. Patient Data

This study analyzed data from a total of 131 subjects, including 29 Ctrl subjects, 56 patients with AD, and 46 patients with Pso. The demographic and electrophysiological characteristics of the study groups are summarized in Table 1.

Table 1 presents the distribution of patients within each group based on age, severity of the condition measured using the 5-point IGA score, and disease duration.

In the Ctrl group, a total of 29 subjects were included. The mean age of the Ctrl group was 31.8 ± 11.1 years. Further analysis within this group revealed that 21 subjects were below the age of 40, with a mean age of 25.52 ± 4.2 years. The remaining eight subjects were above the age of 40, with a mean age of 48.25 ± 4.27 years. The severity of the condition and disease duration were not applicable to the control group and were thus not reported.

In the AD group, a total of 56 patients were included. The mean age of the AD group was 34.5 ± 15 years. Among the patients with AD, 37 individuals were below the age of 40, with a mean age of 24.89 ± 4.62 years. The remaining 19 patients were above the age of 40, with a mean age of 53.32 ± 8.86 years. The severity of the condition, as measured by IGA, was 2.7 ± 1.1 for the entire AD group, indicating a moderate level of disease severity. The mean disease duration for the AD group was 14.1 ± 11.8 years.

In the Pso group, a total of 45 patients were included. The mean age of the Pso group was 53.4 ± 15.7 years. Among the patients with psoriasis, nine individuals were below the age of 40, with a mean age of 30.11 ± 6.92 years. The remaining 36 patients were above the age of 40, with a mean age of 59.2 ± 11.1 years. The severity of the condition, as measured by IGA, was 2.9 ± 1 for the entire Pso group, indicating a moderate level of disease severity. The mean disease duration for the Pso group was 13.2 ± 12.2 years.

These findings highlight the demographic characteristics and clinical parameters of the study groups. The Ctrl group consisted of individuals without any reported disease severity or duration. In contrast, both the AD and Pso groups showed a wide age range, with varying levels of disease severity and disease duration. These results provide a foundation for the further analysis and interpretation of the study’s findings. 

### 3.2. Electrophysiological Data

The analysis of the HRV revealed that the PNS index was lower in the AD group than that in the Ctrl group. Furthermore, we observed higher SNS in the psoriatic group compared with the AD and Ctrl groups. This was further supported by our findings of significant differences in the resistance values on the right side of the peripheral skin compared with the Ctrl and AD groups. These results are consistent with the previously observed right hemispheric frontal EEG bias (Table 2 and Table 3).

The main findings from the EEG data after the post hoc tests show that relative prefrontal activity was more right-lateralized in the AD group compared with the Ctrl group (*p* < 0.001) (Table 3). Additionally, the prefrontal activity was right-lateralized, but to a lesser degree (*p* = 0.046), in the case of the psoriatic patient group.

**Figure 1:**
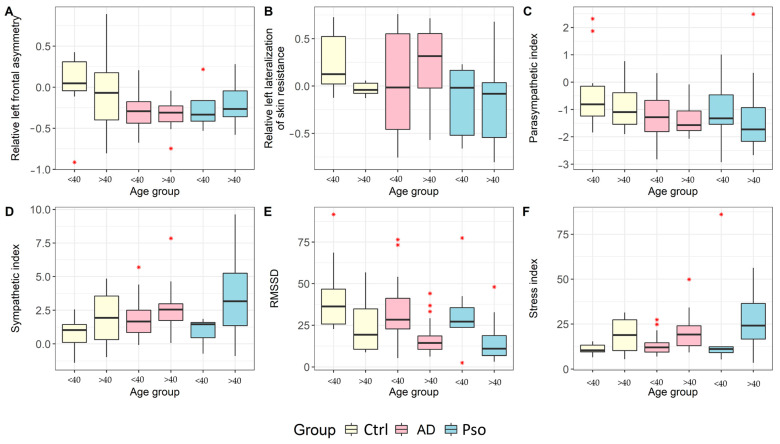
Differences between healthy control subjects (Ctrl), patients with atopic dermatitis (AD), and patients with plaque psoriasis (Pso). The asterisk (*) indicates outliers. AD: atopic dermatitis; Ctrl: control subject; Pso: plaque psoriasis. (**A**–**F**): Levels of various parameters in younger (<40) and older (>40) individuals, including relative left frontal asymmetry (**A**), relative left lateralization of skin resistance (**B**), parasympathetic index (**C**), sympathetic index (**D**), RMSSD (**E**), and stress index (**F**).

To account for the potential influence of left-handedness on our results, we also performed an alternative analysis that excluded left-handed patients from our sample. Interestingly, this led to a strengthening of the observed differences between the Ctrl group and right-handed patients with Pso and AD, particularly in terms of rLFA. Furthermore, when only right-handed patients were included in the analysis, the difference in the PNS index between the AD and Ctrl groups, which was observed in the total sample, disappeared. Significantly, the other findings related to rLFA and PNS in the right-handed group did not differ from those in the full sample (see Table 4 and Figure 1). These results suggest that left-handedness may have been associated with a confounding effect on our analysis, and that right-handed patients primarily drove the observed differences in our sample.

The results showed a significant difference in the PNS index between the Ctrl group and patients with AD (*p* = 0.027), but not between the two patient groups. Similarly, there was a significant difference in the PNS index between the Ctrl group and Pso patients (*p* = 0.014), but not between the two patient groups. The RMSSD did not reach statistical significance, but age was a significant factor (Table 1). Participants under the age of 40 had higher RMSSD compared with those over the age of 40 (*p* < 0.0001).

The SNS and the stress index were higher in older participants than in younger ones (*p* = 0.002 and *p* = 0.003, respectively). Although the main effect of group differentiation was not statistically significant for the SNS or stress indexes, age significantly influenced both indices. Participants under the age of 40 had lower values compared with those over the age of 40. 

### 3.3. Behavioral Activation and Inhibition

**Figure 2:**
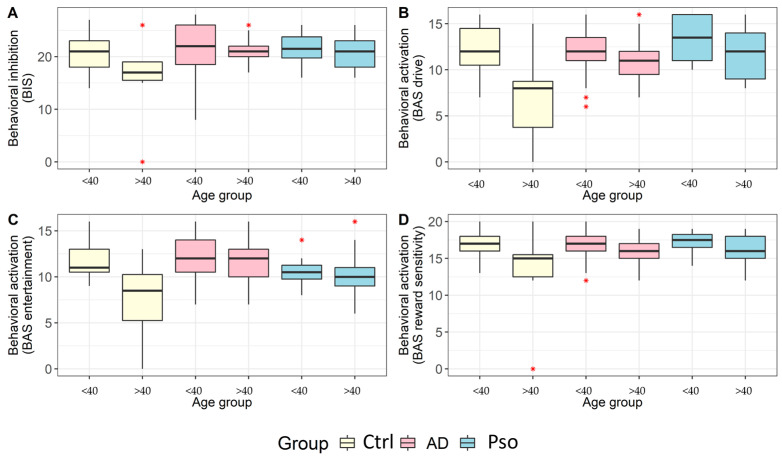
Differences between healthy control subjects, patients with atopic dermatitis (AD), and patients with plaque psoriasis (Pso) in terms of the levels of the behavioral inhibition system (BIS) (**A**), behavioral activation system (BAS) entertainment (**B**), BAS drive (**C**), and BAS reward sensitivity (**D**), categorized by age: younger (<40) and older (>40) individuals.

Upon comparing the different groups, a significant difference was observed between the Ctrl group and patients with AD, indicating that the latter were more inhibited and avoidant. Furthermore, the main effect of group on the BIS scale was statistically significant in the model that included the interaction term (pairwise comparisons: Ctrl subjects were lower than AD patients (*p* = 0.024), Ctrl subjects were lower than Pso patients (*p* = 0.059), and AD patients were equal to Pso patients (*p* = 1)). (Figure 2).

Although a hypothesis linking the right hemispheric predominance to early traumatic experiences has been proposed, the present study did not find significant psychological support for this conjecture. We evaluated this through a battery of standardized questionnaires, including the RSQAA, H-PBI, ACE, and metacognition questionnaires. However, the results only showed the personality features of inhibition and avoidance, as indicated by the BIS and BAS questionnaires. In addition to psychological comparisons, we also examined the impact of age on metacognition using the same statistical tests. No significant differences in metacognition scores were found between the Ctrl group, patients with Pso, and those with AD. During the assessment, participants were asked to rank their performance relative to their peers on a scale of 1–6 at the end of each level. The primary outcome of the tests was short-term memory performance, while metamemory and self-assessment bias were calculated based on the collected data. 

## 4. Discussion

This study aimed to investigate the relationship between frontal hemispherical lateralization associated with different adaptive behavioral patterns and two common inflammatory skin diseases, namely AD and Pso. Previous research has suggested a correlation between emotional stressors, psychiatric disorders, and skin diseases. A meta-analysis of 35 studies by Xie et al. (2019) found that children and adolescents with AD had a significantly higher risk of mental disorders than those without AD [53]. In addition, Locala et al. demonstrated a link between stressful life events and the onset of skin disease with immunopathological dynamics [54].

We used psychophysiological measurements and psychological tests to compare these groups and found some interesting differences. We also analyzed the results of psychological questionnaires. The questionnaires included the BIS and BAS Scales, the Relationship Scale Questionnaire for Adult Attachment (RSQAA), the H-PBI, and the ACE and metacognition tests. Our analysis revealed that only the BIS and BAS Scales showed statistically significant differences between the groups; there were no significant differences between the groups for the other questionnaires (RSQAA, H-PBI, metacognition, and ACE). We split our participants into two groups based on their age: under 40 years of age and above 40 years of age, because age can influence these variables. The timing of adverse childhood experiences can affect later health [55,56], the BIS/BAS Scales can differ significantly with age [57], and attachments, family, and peer attachments can vary significantly with age; attachment anxiety is the highest among younger adults and the lowest among middle-aged or older adults [58]. 

Our post hoc analysis revealed that the differences in the subscales of the BAS (drive, fun-seeking, and reward sensitivity) were statistically significant for both study groups and age groups, with a significant interaction between the two factors. However, the differences between the groups were only consistent in certain age groups. Specifically, only patients over 40 showed significant differences in BAS scores between the study groups. Further investigation is needed to determine the underlying reasons for this age-related difference and explore the potential compensation mechanisms that patients use. 

In addition, the study found that the activation system worked differently in the Ctrl group and AD and Pso patients. Both avoidance tendencies, BIS, and reward sensitivity were associated with lower PNS activity in the preliminary findings. However, this difference disappeared in the AD group when left-handed patients were excluded. 

The differences in SNS and PNS features, and BIS and BAS scale values suggest that Pso and AD have different psychophysiological mechanisms and diverse backgrounds. However, they share a common right-hemispherical lateralization, to some extent.

However, no significant evidence supported the hypothesis that right hemispherical predominance is associated with early traumatic experiences, based on this study’s RSQAA, H-PBI, and ACE questionnaires. 

This study also investigated the cognitive and metacognitive abilities of the subjects before developing novel psychodermatology-specific processes, as self-awareness and self-reflection are essential for most psychotherapeutic interventions. However, the investigation did not find significant differences in cognition and metacognitive abilities between the Ctrl, AD, and Pso groups. Therefore, further research may be needed to explore existing psychotherapeutic approaches for these groups’ supplementary or direct treatment. 

Frontal asymmetry is often approached as a predictor or consequence variable regarding motivation, emotional regulation, and psychopathology [40]. The degree of relative left-frontal EEG activity (rLFA) was used to determine neurophysiological differences with immunological significance. Our hypothesis that both groups would show rightward hemispheric lateralization was confirmed. As lower frontal asymmetry scores (right minus left alpha) may indicate relatively more activity in the right hemisphere of the cortex, alpha activity is believed to inhibit cortical network activity [41]. In the case of group AD, the more significant relative right-hemispheric-dominant prefrontal activation was associated with higher levels of behavioral inhibition and avoidance (higher BIS score) compared with the Ctrl and Pso groups. Panconesi et al. listed personality features and traits of patients suffering from atopic dermatitis, among which insecurity, tension, anxiety, depression, dependence, sensitivity, difficulty in expressing feelings, and shyness might have been related to higher levels of avoidance and behavioral inhibition [59]. Hashiro and Okumura conducted a study focusing on psychosomatic symptoms in patients with AD. They identified that patients with AD were more depressive than Ctrl subjects [60]. In addition, children with AD had lower social competence and lower stress resilience than healthy children [61], which made patients vulnerable to stressors causing chronic stress. These findings are consistent with the dominant prefrontal activation of AD patients in our study, which maintained a continuous state of alertness and vigilance, protecting individuals from adverse and painful outcomes by increasing avoidance behavior. 

There are many models on the hemispherical differences of emotionality. Sackeim et al. suggested that the right hemisphere processes negative emotions, while the left hemisphere is responsible for developing positive emotions [62]. Individuals with better paternal treatment had a relative left-hemispheric-dominant prefrontal activation. On the contrary, the fearful attachment style was significantly correlated with right-hemispheric-predominant prefrontal activation. Sixty percent of mothers with babies suffering from eczema do not calm their baby when crying, compared with sixteen percent in the Ctrl group, and immature, insecure, anxious, dependent, dismissive, overprotective, and unconsciously aggressive mothers do not provide secure skin-to-skin contact to their babies. The babies of depressed mothers show relative right-hemispheric dominant prefrontal activation [63]. Lewis et al. showed that rLFA that showed a shift toward right-hemispheric-predominant prefrontal activation might reflect the connection between environmental stress and adverse health outcomes; the more an individual’s rLFA is altered by periods of stress, the more negative health outcomes one is likely to experience [64]. Another aspect of Davidson’s model, the prefrontal cortex of the left hemisphere, is responsible for approaching behavior. In contrast, the prefrontal cortex of the right hemisphere is responsible for reserved avoidance behavior [65]. In our findings for the group of AD patients, the rLFA and results of the BIS/BAS questionnaire seemed to support this model. The rightward shift in frontal lateralization might be a trait feature of patients with a genetic or developmental background. It could also result from chronic distress, as glucocorticoids can change hemispheric lateralization, according to Ocklenburg et al. [66]. Among HRV indicators, the PNS significantly differed between Ctrl subjects and AD patients, suggesting that Ctrl subjects had a higher PNS index. However, this difference disappeared when right-handed patients were excluded from the sample. 

This study has certain limitations. The first limitation is the lack of molecular data from our patients and the control group. Future studies could investigate the molecular background of AD and Pso in parallel. Analyzing the connections between psychophysiological factors, the Th1 and Th2 pathways, and cellular-level information would provide an excellent basis for further research and treatment. The last limitation of our study is that this study could not isolate cause-and-effect relationships in time, and we could not determine which was earlier, a hemispheric dominant prefrontal activation or an inflammatory disease. These two could be in a circular causal relationship, reinforcing each other. Further investigations and more profound research are needed to reveal the causal relationships between the skin and the mind. 

Regarding the potential therapeutic benefits, the findings presented in this study significantly contribute to the understanding of the psychological aspects and therapeutic approaches associated with dermatological and inflammatory skin diseases. Many psychological interventions have been tested for atopic dermatitis and psoriasis, including brief dynamic psychotherapy, cognitive–behavioral therapy, habit-reversal behavioral therapy, a stress management program, and structured educational programs, with prosperous results [16,67,68,69,70]. However, most of these approaches are not personalized and mostly empirical. These findings confirm the validity of previous therapeutic treatments and open the potential for personalized treatment. With enough support and the right background, a patient with a chronic illness can be provided the right information to plan personalized psychological therapy.

In conclusion, this study sheds light on the relationship between frontal hemispherical lateralization, psychological factors, and two common inflammatory skin diseases: AD and Pso. The study revealed that patients with AD and Pso exhibited different psychophysiological mechanisms and diverse backgrounds. While both groups share a typical right hemispherical lateralization, only AD patients demonstrated prefrontal activation associated with higher behavioral inhibition and avoidance levels. Furthermore, the study did not find significant evidence that right hemispherical predominance was related to early traumatic experiences. The findings suggest that a deeper understanding of these skin diseases’ psychophysiological mechanisms is necessary to develop more effective treatments. Future research in psychodermatology can benefit from these findings to design more efficient psychotherapeutic interventions that target the psychological factors contributing to the onset and maintenance of chronic inflammatory skin diseases.

## 5. Conclusions

In conclusion, further investigation is needed to understand the psychophysiological mechanisms underlying Pso and AD fully. Our findings suggest the importance of considering the psychosomatic background mechanisms underlying different inflammatory skin diseases. More research is necessary to investigate the relationship between stress and specific inflammatory skin diseases and explore potential interventions to address the psychosomatic factors contributing to disease onset and progression.

## Figures and Tables

**Table 1:** Demographic and electrophysiological characteristics of the study groups.

Group	Patients (*n*)	Age (Year)	Severity (IGA)	Disease Duration (year)	rLFA	PNS	SNS	Stress Index	RMSSD
**Ctrl**	Total (29)	31.8 ± 11.1	-	-	0.009 ± 0.425	−0.57 ± 1.21	1.1 ± 1.61	12.67 ± 6.68	37.4 ± 21
	<40 (21)	25.52 ± 4.2	-	-	0.049 ± 0.356	−0.49 ± 1.26	0.84 ± 1.23	10.82 ± 2.71	40.9 ± 20.3
	>40 (8)	48.25 ± 4.27	-	-	−0.061 ± 0.548	−0.83 ± 1.16	1.94 ± 2.57	18.7 ± 12.1	26 ± 22.2
**AD**	Total (56)	34.5 ± 15	2.7 ± 1.1	14.1 ± 11.8	−0.311 ± 0.221	−1.275 ± 0.711	2.03 ± 1.49	15.36 ± 7.75	27.4 ± 16.1
	<40 (37)	24.89 ± 4.62	2.5 ± 1.1	13.2 ± 9.8	−0.302 ± 0.247	−1.225 ± 0.778	1.77 ± 1.28	12.82 ± 4.68	32.2 ± 16.2
	>40 (19)	53.32 ± 8.86	3.2 ± 0.8	16 ± 15.5	−0.327 ± 0.176	−1.374 ± 0.56	2.55 ± 1.76	20.3 ± 10	17.8 ± 10.8
**Pso**	Total (45)	53.4 ± 15.7	2.9 ± 1	13.2 ± 12.2	−0.216 ± 0.265	−1.36 ± 1.11	3.03 ± 2.58	24.5 ± 16	17.5 ± 14.9
	<40 (9)	30.11 ± 6.92	3.4 ± 0.7	13.6 ± 11.2	−0.244 ± 0.324	−1 ± 1.26	0.957 ± 0.984	19.4 ± 27.1	32.2 ± 23.2
	>40 (36)	59.2 ± 11.1	2.7 ± 1	13.1 ± 12.7	−0.21 ± 0.261	−1.44 ± 1.08	3.46 ± 2.61	25.7 ± 12.4	14.2 ± 10.2

AD: atopic dermatitis; Ctrl: control subject; IGA: Investigator’s Global Assessment; PNS: parasympathetic nervous system; Pso: plaque psoriasis; rLFA: relative left frontal activation; RMSSD: root-mean-square of successive RR interval differences; SNS: sympathetic nervous system.

**Table 2:** Descriptive statistics of electrophysiological data for measures of relative left-frontal activity (rLFA), parasympathetic nervous system (PNS), sympathetic nervous system (SNS), stress index, and root-mean-square of successive differences (RMSSD).

Measure	F	Partial Eta^2	Partial Eta^2 (Age)	Partial Eta^2 (Right-Handed)	*p* Value
**rLFA**	8.067	0.167	0.003	0.166	0.001
**PNS**	4.472	0.071	0.036	0.756	0.014
**SNS**	6.426	0.111	0.179	0.106	0.002
**Stress index**	9.72	0.136	0.164	0.151	0
**RMSSD**	9.471	0.147	0.26	0.151	0

AD: atopic dermatitis; Ctrl: control subject; F: F-statistic; Partial eta^2: measure of effect size; Partial eta^2 (age): age effect size; Partial eta^2 (right-handed): handedness effect size; PNS: parasympathetic nervous system; Pso: plaque psoriasis; rLFA: relative left-frontal activation; RMSSD: root mean square of successive RR interval differences; SNS: sympathetic nervous system.

**Table 3:** Results of pairwise Bonferroni tests conducted for the AD (atopic dermatitis), Pso (plaque psoriasis), and Ctrl (control) groups. Dependent variables include AD and Pso. The table displays each pairwise comparison’s mean difference (I-J), standard error, and significance level. The lower 95% confidence interval bound is also provided for each comparison.

Dependent Variable	Study Group	Reference Group	MD ± SE (I-J)	*p* Value	95% CI
**rLFA**	Ctrl	AD	0.320 ± 0.080	0.000	[0.125; 0.515]
		Pso	0.225 ± 0.091	0.046	[0.003; 0.446]
	AD	Ctrl	−0.320 ± 0.080	0.000	[−0.515; −0.125]
		Pso	−0.096 ± 0.076	0.633	[−0.281; 0.090]
	Pso	Ctrl	−0.225 ± 0.091	0.046	[−0.446; −0.003]
		AD	0.096 ± 0.076	0.633	[−0.090; 0.282]
**PNS**	Ctrl	AD	0.703 ± 0.265	0.027	[0.059; 1.347]
		Pso	0.792 ± 0.275	0.014	[0.124; 1.460]
	AD	Ctrl	−0.703 ± 0.265	0.027	[−1.346; −0.059]
		Pso	0.089 ± 0.197	1.000	[−0.391; 0.568]
	Pso	Ctrl	−0.792 ± 0.275	0.014	[−1.459; −0.124]
		AD	−0.089 ± 0.197	1.000	[−0.568; 0.390]
**SNS**	Ctrl	AD	−0.926 ± 0.545	0.276	[−2.251; 0.399]
		Pso	−1.918 ± 0.565	0.003	[−3.292; −0.545]
	AD	Ctrl	0.926 ± 0.545	0.276	[0.005; 1.847]
		Pso	−0.992 ± 0.406	0.049	[−1.979; −0.005]
	Pso	Ctrl	1.918 ± 0.565	0.003	[0.543; 3.292]
		AD	0.992 ± 0.406	0.049	[0.005; 1.979]
**Stress-index**	Ctrl	AD	−2.688 ± 3.174	1.000	[−10.406; 5.030]
		Pso	−11.653 ± 3.274	0.002	[−19.612; −3.693]
	AD	Ctrl	2.688 ± 3.174	1.000	[−5.030; 10.406]
		Pso	−8.965 ± 2.353	0.001	[−14.685; −3.245]
	Pso	Ctrl	11.653 ± 3.274	0.002	[3.693; 19.612]
		AD	8.965 ± 2.353	0.001	[3.245; 14.685]
**RMSSD**	Ctrl	AD	10.012 ± 4.579	0.093	[−1.125; 21.150]
		Pso	19.965 ± 4.785	0.000	[8.326; 31.604]
	AD	Ctrl	−10.012 ± 4.579	0.093	[−21.150; 1.125]
		Pso	9.953 ± 3.460	0.015	[1.537; 18.369]
	Pso	Ctrl	−19.965 ± 4.785	0.000	[−31.604; −8.326]
		AD	−9.953 ± 3.460	0.015	[−18.369; −1.537]

AD: atopic dermatitis; CI: confidence interval; Ctrl: control subject; MD: mean difference; PNS: parasympathetic nervous system; Pso: plaque psoriasis; rLFA: relative left-frontal activation; RMSSD: root-mean-square of successive RR interval differences; *p* value: probability value; SE: standard error; SNS: sympathetic nervous system.

**Table 4:** Results of the pairwise Bonferroni test without including data from left-handed patients.

Dependent Variable	Study Group	Reference Group	MD ± SE (I-J)	*p* Value	95% CI
**rLFA**	Ctrl	AD	0.330 ± 0.084	0.001	[0.125, 0.536]
		Pso	0.231 ± 0.094	0.048	[0.001, 0.462]
	AD	Ctrl	−0.330 ± 0.084	0.001	[−0.536, −0.125]
		Pso	−0.099 ± 0.079	0.633	[−0.291, 0.093]
	Pso	Ctrl	−0.231 ± 0.094	0.048	[−0.462, −0.001]
		AD	0.099 ± 0.079	0.633	[−0.093, 0.291]
**PNS**	Ctrl	AD	0.625 ± 0.272	0.071	[−0.038, 1.287]
		Pso	0.787 ± 0.282	0.019	[0.099, 1.474]
	AD	Ctrl	−0.625 ± 0.272	0.071	[−1.287, 0.038]
		Pso	0.162 ± 0.204	1.000	[−0.334, 0.658]
	Pso	Ctrl	−0.787 ± 0.282	0.019	[−1.474, −0.099]
		AD	−0.162 ± 0.204	1.000	[−0.658, 0.334]
**SNS**	Ctrl	AD	−0.780 ± 0.532	0.436	[−2.074, 0.514]
		Pso	−1.814 ± 0.552	0.004	[−3.157, −0.471]
	AD	Ctrl	0.780 ± 0.532	0.436	[−0.514, 2.074]
		Pso	−1.034 ± 0.398	0.033	[−2.004, −0.064]
	Pso	Ctrl	1.814 ± 0.552	0.004	[0.471, 3.157]
		AD	1.034 ± 0.398	0.033	[0.064, 2.004]
**Stress-index**	Ctrl	AD	−2.107 ± 3.196	1.000	[−9.888, 5.674]
		Pso	−10.394 ± 3.295	0.006	[−18.417, −2.372]
	AD	Ctrl	2.107 ± 3.196	1.000	[−5.674, 9.888]
		Pso	−8.287 ± 2.382	0.002	[−14.086, −2.488]
	Pso	Ctrl	10.394 ± 3.295	0.006	[2.372, 18.417]
		AD	8.287 ± 2.382	0.002	[2.488, 14.086]
**RMSSD**	Ctrl	AD	8.272 ± 4.755	0.255	[−3.307, 19.851]
		Pso	19.200 ± 4.974	0.001	[7.087, 31.312]
	AD	Ctrl	−8.272 ± 4.755	0.255	[−19.851, 3.307]
		Pso	10.928 ± 3.618	0.010	[2.116, 19.739]
	Pso	Ctrl	−19.200 ± 4.974	0.001	[−31.312, −7.087]
		AD	−10.928 ± 3.618	0.010	[−19.739, −2.116]

AD: atopic dermatitis; CI: confidence interval; Ctrl: control subject; MD: mean difference; PNS: parasympathetic nervous system; Pso: plaque psoriasis; rLFA: relative left-frontal activation; RMSSD: root-mean-square of successive RR interval differences; Sig.: significance; SE: standard error; SNS: sympathetic nervous system.

## Data Availability

The data supporting this study’s findings are available from the corresponding author S.B. upon reasonable request.
